# Cognitive Impairment in Adult Attention Deficit Hyperactivity Disorder: Clinical Implications and Novel Treatment Strategies

**DOI:** 10.3390/clinpract15080150

**Published:** 2025-08-12

**Authors:** Alexis J. Vega, Gabriel V. Hernandez, Ahmed I. Anwar, Bahareh Sharafi, Rahib K. Islam, Sahar Shekoohi, Alan D. Kaye

**Affiliations:** 1 School of Medicine, Tulane University, 1430 Tulane Ave, New Orleans, LA 70112, USA; 2 Department of Psychology, Quinnipiac University, 275 Mount Carmel Ave, Hamden, CT 06518, USA; 3 Psychiatry Department, Citrus Health Herbert Wetheim College of Medicine, 4175 W 20th Ave, Hialeah, FL 33012, USA; 4 School of Medicine, Louisiana State University Health and Sciences Center in New Orleans, New Orleans, LA 70112, USA; 5 Department of Anesthesiology, Louisiana State University Health Sciences Center at Shreveport, Shreveport, LA 71103, USA

**Keywords:** adult deficit hyperactivity disorder (ADHD), cognition, adults, neurofeedback, viloxazine, modafinil, cognitive behavioral therapy (CBT), dialectical behavioral therapy (DBT)

## Abstract

Attention Deficit Hyperactivity Disorder (ADHD) is a lifelong condition; however, traditional treatment focuses on hyperactivity and inattention, which is largely a manifestation of pediatric ADHD. Studies are limited regarding cognitive difficulties, as seen in adult ADHD, as well as treatment strategies for this population. This review of the literature examines multiple recent studies that discuss various novel treatment strategies for cognitive impairment in adults with ADHD. A targeted literature review was conducted using PubMed to identify recent studies on cognitive dysfunction in adults with ADHD, with an emphasis on emerging treatment strategies. Data collected included sample size, intervention strategies, cognitive function, and side effects. Studies on non-invasive brain stimulation revealed significant effects on executive function in adult ADHD patients. Other studies revealed statistically significant improvements in cognitive flexibility and response inhibition in modafinil users. Another study demonstrated significant improvement in working memory with off label use of viloxazine for adults. This review of the literature describes the effectiveness of novel treatment strategies of adult ADHD including non-stimulant medications, behavioral therapies and neurofeedback. This highlights the need for treatment modalities that enhance cognitive outcomes and further research into long-term efficacy and safety of these novel interventions and implementing psychological treatment into medical management of adult ADHD.

## 1. Introduction

Attention Deficit Hyperactivity Disorder (ADHD) is a neurodevelopmental condition that is historically diagnosed in childhood, but its impact frequently extends into adulthood [1]. While ADHD is traditionally associated with inattention and hyperactivity, growing evidence describes its significant impact on adult cognitive function, particularly in executive function, working memory, and cognitive flexibility [2]. These impairments can substantially disrupt occupational performance, academic success, and overall quality of life [3].

A 2023 study by Isaac et al. defined ADHD using two theoretical frameworks. One framework, the executive dysfunction theory, defines executive functions as the top-down cognitive processes underlying goal-directed behavior, including inhibitory control, working memory, and additional guidance, and state-regulation theory, which emphasizes the role of arousal in cognitive efficiency [3].

A 2016 meta-analysis by Cortese et al. reports that up to 60% of ADHD children continue to experience symptoms into adulthood [4]. A 2021 study by Butzbach et al. demonstrates that ADHD in adulthood presents with executive dysfunction and cognitive impairment more often compared to childhood ADHD [2].

Despite the prevalence of cognitive dysfunction in adults with ADHD, current pharmacological treatments primarily target behavioral symptoms and often neglect cognitive domains [5,6]. A 2023 study by Zhang et al. highlights that while traditional pharmacological treatments for adult ADHD mainly target behavioral symptoms and often overlook cognitive impairments, emerging remote neuro-cognitive interventions, such as cognitive training, EEG neurofeedback, and transcranial electrical stimulation are being developed to specifically address cognitive deficits in this population [5].

This review of the literature evaluates emerging treatment strategies specifically targeting cognitive dysfunction in adults with ADHD. By examining recent findings, our study aims to inform more individualized, cognition-focused care approaches that can enhance functional outcomes and quality of life for this population.

## 2. Methods

A literature review was conducted using PubMed, focusing on peer review studies related to Attention-Deficit/Hyperactivity Disorder and cognitive function in adults. Keywords included combinations of “ADHD,” “adult,” “cognition,” “executive function,” “cognitive impairment,” and “treatment.” The search was limited to articles published between 2013 and 2025 to prioritize recent evidence and emerging treatment strategies. Earlier references were also included to provide context on current clinical manifestations and standard treatment approaches. Inclusion criteria were original research articles and systematic reviews investigating the cognitive profile of adults with ADHD and interventions targeting cognitive dysfunction.

## 3. The ASRS: A Reliable Tool for Identifying Adult ADHD

Accurately diagnosing ADHD in adults presents unique challenges, as symptoms often overlap with other psychiatric conditions that may manifest differently than in childhood. Unlike pediatric assessments, adult ADHD diagnosis relies heavily on self-reported experiences, retrospective evaluation of childhood behavior, and the exclusion of alternative explanations. As a result, the use of validated screening tools like the Adult ADHD Self- Report Scale (ASRS) has become increasingly important in helping clinicians identify individuals who may require further diagnostic evaluation [6].

A 2020 study by Anbarasan et al. evaluated the effectiveness of the ASRS and the Wender Utah Rating Scale (WURS) in distinguishing adults with ADHD from those without. In a sample of over 1500 participants, including previously diagnosed ADHD patients, the full 18-item ASRS and the abbreviated 6-item screening tool demonstrated excellent accuracy, with area under the curve (AUC) values of approximately 0.90. The WURS, which measured retrospective childhood symptoms, showed even higher accuracy, and when used in combination with the ASRS, the overall screening precision improved further. These findings suggest the ASRS’s accuracy in identifying patients who will likely meet the criteria for adult ADHD [6].

Brevik et al. (2020) assessed the validity of the ASRS in a Norwegian population, comparing 646 adults with ADHD to 908 population controls [7]. Similar to Anbarasan et al., their findings confirmed high sensitivity and specificity for both the full and short versions of ASRS, with AUC 0.956 (95% CI: 0.946–0.965) for the WURS and 0.904 (95% CI: 0.888–0.921) for the ASRS. Factor analysis supported the scale’s structure, and results demonstrated that the ASRS is both time-efficient and psychometrically sound [7].

Together, these studies provide strong evidence that the ASRS is a reliable and accurate tool for identifying adults who may have ADHD, and it serves as a valuable first step in the diagnostic process when followed by a comprehensive evaluation.

## 4. Cognitive Deficits Studied in Adult ADHD

While the exact causes of ADHD are not fully known, research suggests that there are specific cognitive deficits associated with the disorder. These deficits primarily involve executive functions, which are cognitive processes that help regulate behavior, control impulses, and plan and organize tasks. Specifically, the deficits concern inattention, cognitive flexibility, inhibition and impulsivity, and working memory. These difficulties can significantly affect daily decision making, interpersonal functioning, and long-term goal achievement. Eberhard et al. (2024) conducted a neuropsychological study evaluating cognitive deficits in young adult psychiatric patients with ADHD using the WAIS-IV and D-KEFS batteries to assess intellectual functioning and executive skills [8]. The study consisted of 141 young adult psychiatric patients (78 who had been previously diagnosed with ADHD). The ADHD group demonstrated significantly lower verbal comprehension and full-scale intelligence quotient compared to the non-ADHD group. However, tests measuring working memory or executive function did not differentiate between the two groups, suggesting that while certain cognitive domains are impacted, others may not be as affected in ADHD adults [8].

A 2022 study by Hong et al. compared cognitive developmental trajectories between 240 adults with ADHD and 244 neurotypical controls using longitudinal and cross-sectional assessments. Adults with ADHD showed delayed maturation in executive functions, particularly into adulthood, suggesting a slower or altered cognitive developmental path rather than complete normalization over time. The findings highlight the need for ongoing support and interventions targeting cognitive function in adults with ADHD [9]. Deficits in working memory and inattention experienced by adults with ADHD can significantly impair daily functioning, leading to difficulties in time management, task completion, and maintaining focus in work or social settings. These difficulties can reduce quality of life by increasing stress, lowering productivity, and straining personal relationships [10].

Cognitive flexibility, defined as the ability to shift attention, adapt strategies, and switch between tasks or mental sets, is often impaired in ADHD adults. A 2018 empirical study by Luna-Rodriguez et al. examined cognitive flexibility in 38 adults with ADHD and 39 matched controls using a task-switching paradigm involving hierarchical (Navon) stimuli. By manipulating task-switching and attentional set-shifting demands independently, they found that adults with ADHD showed slower and more variable responses, specifically on trials requiring shifts in attentional focus, suggesting impaired cognitive flexibility. The study concluded that ADHD is associated with a deficit in flexible deployment of attention to varying sources of stimulus information [11].

Similarly, Guo et al. (2023) assessed 319 adults (173 with ADHD, 146 without) using a comprehensive neuropsychological battery and applied network analysis to explore interrelations among cognitive functions [12]. A denser network with stronger global connectivity was observed in the ADHD group compared to the non-ADHD group. Their results highlighted cognitive flexibility as a central deficit in adults with ADHD, strongly interconnected with processing speed and fluency, indicating its critical role in the broader neuro-cognitive profile of the disorder [12]. Cognitive flexibility deficits in ADHD can lead to inflexible problem solving and difficulty adapting to novel or complex tasks. These impairments are often linked to poor response inhibition, an aspect of inhibitory control, which contributes to impulsivity and the inability to suppress automatic or impulsive behavior [13].

Recent neuroimaging studies have advanced our understanding of neurobiology and anatomy underlying cognitive function in adults with ADHD. For example, a voxel-wise mega-analysis by Norman et al. in 2024 revealed that ADHD patients showed greater connectivity between subcortical regions and cortical areas, particularly involving bilateral caudate connectivity [14]. The results support a role for subcortico-cortical circuits in ADHD but indicate that these neural features explain only a small portion of ADHD’s overall pathophysiology [14].

Another 2023 study by Li et al. examined working memory-related brain activation in 362 ADHD children using fMRI during a two-back task, comparing typically developing children (TDC), children with ADHD and no family history (ADHD-NF), and those with a family history of ADHD (ADHD-F). Both ADHD groups and the ADHD-F groups showed reduced activation in the left inferior frontal gyrus compared to TDC. These findings suggest distinct functional alterations in the IFG in ADHD, highlighting potential neural markers for diagnosis and intervention [15].

A 2023 study by Misra et al. investigated dynamic functional connectivity (dFC) in ADHD patients using resting-state fMRI. The researchers found significantly higher variability in dFC across multiple brain networks in the ADHD group. Notably, increased instability was observed in the temporal gyri, anterior cingulate, and right parietal regions. The findings suggest that functional connectivity instability may underlie core attentional symptoms in ADHD [16].

Parlatini et al. (2023) conducted a systematic review and meta-analysis of 129 diffusion-weighted imaging studies, finding reduced fractional anisotropy in the corpus callosum and cingulum, especially in adults with ADHD [17]. These white matter changes were associated with cognitive deficits and symptom severity. However, methodological limitations in many studies highlight the need for better imaging protocols and consistency in future research [17].

## 5. Current Management of Adult Attention Deficit Hyperactivity Disorder

The current treatment of ADHD in adults involves a comprehensive approach that combines medication and behavioral therapy. A 2005 study by Safren and Beiderman evaluated the effectiveness of cognitive-behavioral therapy (CBT) for adults with ADHD who were already on stable medication but continued to experience significant symptoms. Thirty-one participants were randomly assigned to either CBT plus medication or medication alone. Results showed that those receiving CBT had significantly greater reductions in ADHD symptoms, anxiety, and depression, based on both clinician and self-reports. Over half (56%) of the CBT group were classified as treatment responders, compared to only 13% in the medication-only group. These findings indicate that combining CBT with stimulant medication is more effective than medication alone in reducing core ADHD symptoms and associated emotional difficulties. The results support CBT as a feasible and promising adjunct treatment for adults with residual ADHD symptoms [18].

While treatment for ADHD in adults shares similarities with pediatric approaches, important differences must be considered. Stimulants like methylphenidate (Ritalin) and amphetamines (Adderall) are effective in managing symptoms such as inattention and impulsivity in both groups, but adult dosing requires individualized adjustments [19].

Non-stimulant options like atomoxetine (Strattera) and bupropion (Wellbutrin) are valuable alternatives, especially for those who cannot tolerate stimulants or are at risk for misuse [20].

Atomoxetine, FDA-approved for children and adults, acts as a norepinephrine reuptake inhibitor, increasing both norepinephrine and dopamine levels in the prefrontal cortex (where dopamine transmitters are scarce) by targeting norepinephrine transporters [21,22,23].

Durell et al. (2013) conducted a 12-week, double-blind, placebo-controlled trial with 220 adults ages 18–30 to evaluate atomoxetine for ADHD [23]. The authors measured ADHD symptoms and treatment effects using Conner’s Adult ADHD Rating scales, (CAARS-Inv:SV) the Adult ADHD Quality of Life Questionnaire (AAQoL), and the Behavior Rating Inventory of Executive Function-Adult Version (BRIEF-A). The atomoxetine group showed significantly greater improvements in ADHD symptoms, quality of life, and executive function compared to placebo. The treatment was generally well tolerated, with common side effects including nausea and insomnia [23].

In a 7-week, double-blind, placebo-controlled crossover trial by Spencer et al. in 2001, 27 adults with ADHD were treated with methylphenidate and Adderall, each for three weeks, separated by a one-week placebo period. Methylphenidate led to a 32% reduction (*p* < 0.001) in ADHD symptom severity, with 60% of participants meeting response criteria (≥30% symptom reduction and a CGI improvement score ≤ 2) [24]. Adderall produced a 42% symptom reduction (*p* < 0.001) with a higher response rate of 70%. Both medications significantly outperformed placebo (7% response rate) and were well tolerated, with common side effects including insomnia and decreased appetite. Although Adderall showed slightly greater efficacy, the study found no statistically significant difference between the two treatments [24].

Methylphenidate primarily works by blocking dopamine and norepinephrine transporters, increasing their levels in the brain to improve attention and executive function. Adderall, a mixed amphetamine salt, not only blocks these transporters but also promotes the release of dopamine, norepinephrine, and serotonin, resulting in a more potent effect on neurotransmission [18,25].

In addition to medication, psychotherapy or counseling is another important component of ADHD treatment in both adults and children. Specifically, cognitive behavioral therapy (CBT) has been shown to be effective in addressing specific challenges associated with ADHD, such as time management, organization, and emotional regulation [18]. A 2010 randomized controlled trial by Safren et al. evaluated the efficacy of CBT for 86 adults with ADHD who were already on medication but still experiencing significant symptoms. Over 12 weekly sessions, participants received CBT focused on skills like organization, time management, and cognitive restructuring. Compared to a control group receiving relaxation training, the CBT group showed significantly greater reductions in ADHD symptoms and functional impairment, demonstrating CBT’s effectiveness as an adjunct treatment for adult ADHD [18].

Solanto et al. (2010) conducted a randomized controlled trial involving 88 adults with ADHD, administering meta-cognitive therapy, which uses CBT principles and is focused on time management and organizational skills [26]. Efficacy was assessed using the Conners’s Adult ADHD Rating Scale (CAARS), clinician assessments, and executive function measures including the Behavioral Rating Inventory and Executive Function-Adult Version (BRIEF-A). Participants receiving cognitive therapy showed significant reductions in inattentive symptoms and improvements in executive functioning compared to waitlist controls, sustained at 6-month follow-up [26].

## 6. Dialectical Behavioral Therapy as a Novel Therapeutic Strategy for ADHD in Adults

Recent research indicates that short-term, structured therapies like CBT and DBT (Dialectical behavioral therapy) are effective in alleviating ADHD symptoms and improving quality of life. CBT has been used to improve executive functions, such as planning and organization, while DBT focuses on mindfulness and skills to manage relationships, self-esteem, impulsiveness, and consequence thinking. Combining elements from both CBT and DBT manuals could provide a more comprehensive treatment for the diverse issues associated with ADHD than CBT alone. A study by Nasri et al. in 2020 explored a new combined CBT/DBT for adults with ADHD and found it to be feasible and effective [27]. A total of 18 participants completed a 14-session program and self-reported ADHD symptom scales (ASRS) with strong improvements in core ADHD symptoms and associated emotional difficulties. They delivered 14 weekly group sessions (approximately 3.5 months total) and followed participants up to 6 months post-treatment. The most notable improvements were in inattention, leading to enhanced executive functioning and working memory, and these reduction in symptoms were sustained at the 6-month follow-up indicating some lasting benefits. Compared to previous psychotherapy studies for ADHD, which had response rates of 32–67%, this approach achieved a higher proportion of responders, with additional benefits including decreased depression, psychological stress, and everyday difficulties [27].

Expanding on the use of behavioral therapy as a treatment strategy for adults with ADHD, Nasri et al. performed an additional randomized control trial in 2023 [28]. The trial compared internet-based cognitive behavioral therapy (iCBT) to an active control treatment of internet-based applied relaxation training (iART) and to treatment as usual (TAU) in adults with ADHD. The study utilized 104 outpatients, 67% who used ADHD medication, and were randomized to 12 weeks of iCBT, iART or TAU. ASRS for iCBT and iART demonstrated significant improvement at post-treatment and 3-month follow-up than for TAU alone, which extended for over 12 months. ASRS were similar between iCBT and iART treatment groups. The findings of the study revealed that iCBT and iART could enhance functioning in adult ADHD when added to regular medical treatment. The study concluded that increasing availability of psychological resources is needed for adult patients with ADHD [28].

## 7. Neurofeedback as a Novel Treatment for Adult ADHD

Neurofeedback is an emerging treatment for adult ADHD that uses a brain–computer interface to help individuals learn to self-regulate brain activity. It involves recording brain signals, processing them in real time, and providing feedback to the user to encourage targeted changes. Based on operant conditioning and neuroplasticity principles, this method aims to enhance cognitive and behavioral functions and is used therapeutically for performance enhancement and in research [29].

One specific neurofeedback approach is Theta/Beta Ratio (TBR) neurofeedback, which targets the reduction in theta and/or increase in beta power in central and frontal brain regions. This protocol addresses common electrophysiological abnormalities in ADHD. Enriquez-Geppert et al.’s 2019 article reviewed findings from numerous large-scale randomized controlled trials and meta-analyses that evaluated neurofeedback protocols [29]. Studies addressed in the paper revealed that 31 to 40 sessions of TBR neurofeedback can be as effective as methylphenidate in academic performance, inattention, and hyperactivity. Slow Cortical Potential (SCP) neurofeedback, typically involving about 35 sessions, focuses on self-regulation of cortical activation and inhibition, specifically targeting shifts between electrical positivity and negativity. This method, associated with improved reaction time, stimulus detection, and short-term memory, is centered on the vertex and differs from TBR and SMR protocols by addressing both activation and inhibition. SCP neurofeedback may also benefit sleep by influencing the sleep spindle circuit, facilitating the transition from wakefulness to sleep, as suggested by Lubar and Shimizu [29].

A 2022 4-week-long randomized, double-blind, parallel, sham-controlled clinical trial by Leffa et al. (TUNED trial) explored the efficacy and safety of home-based transcranial direct current stimulation (tDCS) in 64 stimulant-free adults with ADHD. The participants were randomized to receive 30 min daily home tDCS sessions over four weeks, or sham stimulation. Response was measured through ASRS and cognitive function was assessed with tests targeting attention and executive functions including Continuous Performance Tasks (CPTs) and similar computerized attention measures. Mild adverse events were more frequent in the tDCS group such as skin redness, headache, and scalp burn. The study demonstrated significant improvements in inattention symptoms compared to sham, suggesting that this non-invasive neuromodulation technique could be an effective non-pharmacological option for adults with ADHD [30].

## 8. Novel Medications for Adult ADHD

A 2022 six-week long randomized controlled trial by Nasser et al. investigated the efficacy and safety of viloxazine extended-release in 374 adults with ADHD. Viloxazine is a non-stimulant, norepinephrine/serotonin reuptake inhibitor. In the study, adults with ADHD were randomly assigned to receive either viloxazine or a placebo with outcome measures being improvement in ADHD symptoms by Adult ADHD Investigator Symptom Rating Scale (AISRS) and Clinical Global Impressions (CGI) and Behavioral Rating Inventory of Executive Function-Adult Version (BRIEF-A) to assess executive function. Viloxazine extended-release significantly reduced ADHD symptoms compared to the placebo, with a greater decrease in AISRS total scores (−15.5 vs. −11.7, *p* = 0.0040) and improvements in CGI-S (−1.4 vs. −1.0, *p* < 0.0023). Significant improvements were also observed in inattention, hyperactivity/impulsivity, executive functioning, and global impression ratings by week 2. Viloxazine ER was generally well tolerated, though 9.0% of participants discontinued due to adverse events, most commonly insomnia, fatigue, nausea, and decreased appetite [31].

Furthermore, a 2023 study by Alam and Choudhary investigated the neurochemical effects of modafinil and methylphenidate in data collected from rats, using a sample size of *n* = 12 per group in their behavioral and biochemical experiments. The study utilized Morris Water Maze tests for spatial learning and memory and passive avoidance tests for learning and memory to assess cognition in rats. The study demonstrated that both treatments improved cognitive performance, particularly in stress-induced tasks. The study also showed that modafinil increased brain dopamine and serotonin levels, which may suggest its potential utility in treating stress-related cognitive impairments, with possible relevance for ADHD [32]. These studies highlight the potential of diverse interventions, including medication and non-invasive brain stimulation, in improving cognitive and behavioral outcomes for individuals with adult ADHD. These studies support the findings of the current study and expand the therapeutic landscape, demonstrating that multimodal approaches can provide meaningful cognitive improvements (Table 1).

## 9. Discussion

This literature review underscores the underrecognized cognitive deficits in adult ADHD and evaluates both traditional and emerging treatments targeting these impairments. Although up to 60% of children with ADHD continue to show symptoms in adulthood, most research remains focused on pediatric populations and core symptoms like inattention and hyperactivity [25]. However, deficits in executive function, working memory, and cognitive flexibility are more prominent and disabling in adults, highlighting the need for adult-focused cognitive interventions [2].

Recent research outlined in our study highlights the effectiveness of both pharmacological and non-pharmacological treatments in managing adult ADHD. CBT, including structured and internet-based formats, has been shown to improve executive function and reduce symptoms, with studies by Safren et al., Solanto et al., and Nasri et, al (2023) demonstrating sustained benefits in attention, organization, and emotional regulation [18,26,28]. Emerging strategies like dialectical behavioral therapy (DBT), neurofeedback, and non-invasive neuromodulation techniques such as transcranial direct current stimulation (tDCS) also show promise in improving attention, working memory, and self-regulation. Pharmacological treatments, including stimulants (Adderall and methylphenidate), non-stimulants like viloxazine, and cognitive enhancers like modafinil, continue to demonstrate robust symptom control and cognitive benefits in randomized controlled trials as referenced in trials such as in Nasser et al. 2022 [31]. Together, these findings suggest that a multimodal approach, combining medication with structured behavioral therapies may offer the most comprehensive and lasting improvements in cognitive and functional outcomes for adults with ADHD.

## 10. Limitations

Despite these encouraging developments, several limitations remain. Many of the cited studies, including those on viloxazine, iCBT, and neurofeedback, rely on relatively small sample sizes, limiting generalizability. Additionally, the long-term safety and efficacy of newer modalities, such as neurofeedback and non-invasive brain stimulation, remain unclear. Concerns around the side effects of atomoxetine, such as cardiovascular risks or rare behavioral changes, also warrant careful consideration, especially given the complex comorbidity of adult ADHD [21]. Most therapies are also resource-intensive, require frequent sessions, and may be less accessible to individuals from underserved communities. Furthermore, genetic interventions are still largely theoretical and lack real-world clinical application at this stage [33].

Future trials should explore the effectiveness of combined treatments, such as neurofeedback, CBT/DBT, and non-stimulant medications, in addressing cognitive symptoms of adult ADHD. Additionally, studies comparing gender differences in treatment response could provide valuable insights into personalized care strategies and help address the diverse needs of adults with ADHD. Future research should also focus on large, diverse longitudinal studies to assess long-term efficacy and safety of new treatments, while addressing ADHD’s heterogeneity.

Identifying genetic predisposition of patients who exhibit improved cognitive response to therapeutic intervention can be utilized to further enhance treatment [4,33]. Genetic research has deepened our understanding of ADHD, with genetic factors accounting for 70–80% of the risk. Key genes such as DRD4, DAT1, NET1, and SNAP-25 are linked to dopamine and norepinephrine regulation, and GWAS studies have identified loci tied to neurotransmitter and neurodevelopmental pathways. Polygenic risk scores now offer a promising approach for predicting individual ADHD susceptibility [34,35]. Precision medicine, including genetic screening, could help tailor therapies, and integrated care models combining medication, behavior therapy, and digital tools may improve adherence and outcomes [33].

## 11. Conclusions

This review highlights the critical need to address cognitive deficits in adult ADHD, an area often overshadowed by a pediatric focus on core symptoms. Evidence supports the efficacy of both pharmacological and behavioral interventions in improving executive function, attention, and emotional regulation. While emerging treatments like neurofeedback, tDCS, and DBT show promise, more rigorous large-scale studies are needed to confirm their long-term benefits [36,37]. Further research should focus on personalized multimodal strategies that integrate genetics, behavioral therapy, and digital tools to better target the diverse cognitive challenges faced by adults with ADHD. This will serve to improve patient quality of life and enhance long-term patient outcomes [38].

## Figures and Tables

**Table 1:** Recent studies reviewing novel treatment strategies of cognitive dysfunction in adulthood ADHD [27,28,29,30,31,32].

Study	Groups Studied and Interventions	Results and Findings	Conclusions
Alam et al. (2023) [32]	Rats exposed to stress-induced cognitive deficits; intervention with modafinil and methylphenidate treatments.	Both modafinil and methylphenidate improved cognitive performance in stress-induced tasks and portrayed increased brain dopamine and serotonin levels, with modafinil showing distinct neurochemical effects.	Modafinil may offer cognitive benefits in treating stress-related cognitive impairments, with implications for ADHD treatment.
Nasser et al. (2022) [31]	Adults with ADHD; intervention with viloxazine ER targeting sustained attention and executive function.	Significant Improvements in sustained attention and executive function in adults.	Highlights novel monoaminergic targets beyond dopamine/norepinephrine.
Leffa et al. (2022) [30]	Adults (18–60 yrs) with ADHD with moderate to severe inattention; intervention with 2 mA anodal tDCS over right DLPFC, cathodal over left DLPFC; 20 min/day for 4 weeks.	Significant reduction in inattention for the active tDCS group.	Reinforces the value of non-pharmacological brain stimulation and provides evidence that home-based tDCS is effective and well-tolerated for reducing inattention in adults with ADHD.
Nasri et al. (2020) [27]	18 adults with ADHD from neuropsychiatric units in Stockholm underwent a 14-week program including CBT and DBT.	ADHD symptoms significantly decreased (d = 1.29) and remained stable for 6 months.	Combination CBT/DBT treatments may be a valuable addition to available ADHD treatments in psychiatric care.
Enriquez-Geppert (2019) [29]	Review of the literature based on meta-analyses and large multicenter randomized controlled trials.	Standard neurofeedback training protocols, theta/beta (TBR), sensori-motor rhythm (SMR), and slow cortical potential (SCP) have been shown to be efficacious and adults with ADHD in multiple studies.	Neurofeedback according to the standard protocols in ADHD need to be considered as a viable treatment alternative and further research is warranted to understand neurofeedback protocols.
Nasri et al. (2023) [28]	104 cases, of which 67% used ADHD medication, were randomized to 3 months of iCBT, iART, or TAU.	ASRS improved more for iCBT and iART (at 3-month follow-up) than for TAU.	iCBT and iART could both be promising add-ons to medication.

## Data Availability

Data sharing is not applicable to this article as no datasets were generated or analyzed during the current study.

## References

[1] Shaw P., Eckstrand K., Sharp W., Blumenthal J., Lerch J.P., Greenstein D., Clasen L., Evans A., Giedd J., Rapoport J.L. (2007). Attention-deficit/hyperactivity disorder is characterized by a delay in cortical maturation. Proc. Natl. Acad. Sci. USA.

[2] Butzbach M., Fuermaier A.B.M., Aschenbrenner S., Weisbrod M., Tucha L., Tucha O. (2021). Metacognition in adult ADHD: Subjective and objective perspectives on self-awareness of cognitive functioning. J. Neural Transm..

[3] Isaac V., López V., Escobar M.J. (2024). Arousal dysregulation and executive dysfunction in attention deficit hyperactivity disorder (ADHD). Front. Psychiatry.

[4] Cortese S., Ferrin M., Brandeis D., Holtmann M., Aggensteiner P., Daley D., Santosh P., Simonoff E., Stevenson J., Stringaris A. (2016). Neurofeedback for Attention-Deficit/Hyperactivity Disorder: Meta-Analysis of Clinical and Neuropsychological Outcomes From Randomized Controlled Trials. J. Am. Acad. Child Adolesc. Psychiatry.

[5] Zhang D.-W., Johnstone S.J., Sauce B., Arns M., Sun L., Jiang H. (2023). Remote neurocognitive interventions for attention-deficit/hyperactivity disorder—Opportunities and challenges. Prog. Neuro-Psychopharmacol. Biol. Psychiatry.

[6] Anbarasan D., Kitchin M., Adler L.A. (2020). Screening for Adult ADHD. Curr. Psychiatry Rep..

[7] Brevik E.J., Lundervold A.J., Haavik J., Posserud M.B. (2020). Validity and accuracy of the Adult Attention-Deficit/Hyperactivity Disorder (ADHD) Self-Report Scale (ASRS) and the Wender Utah Rating Scale (WURS) symptom checklists in discriminating between adults with and without ADHD. Brain Behav..

[8] Eberhard D., Gillberg C., Billstedt E. (2024). Cognitive functioning in adult psychiatric patients with and without attention-deficit/hyperactivity disorder. Brain Behav..

[9] Hong J.S., Lee Y.S., Han D.H. (2022). Cognitive developmental trajectories in adult ADHD patients and controls: A comparative study. J. Atten. Disord..

[10] Barkley R.A. (2015). Attention-Deficit Hyperactivity Disorder: A Handbook for Diagnosis and Treatment.

[11] Luna-Rodríguez A., Wendt M., Kerner-Auch K., Gawrilow C., Jacobsen T. (2018). Selective impairment of attentional set shifting in adults with ADHD. Behav. Brain Funct..

[12] Guo X., Lin Y., Chen X., Wang Y., Wang X. (2023). Networks of neuropsychological functions in the clinical evaluation of adult ADHD. Assessment.

[13] Nigg J.T. (2013). Attention-deficit/hyperactivity disorder and adverse health outcomes. Clin. Psychol. Rev..

[14] Norman L.J., Sudre G., Price J., Shaw P. (2024). Subcortico-cortical dysconnectivity in attention-deficit/hyperactivity disorder: A voxelwise mega-analysis across multiple cohorts. Am. J. Psychiatry.

[15] Li X., Motwani C., Cao M., Martin E., Halperin J.M. (2023). Working memory-related neurofunctional correlates associated with the frontal lobe in children with familial vs. non-familial attention-deficit/hyperactivity disorder. Brain Sci..

[16] Misra R., Gandhi T.K. (2023). Functional Connectivity Dynamics show Resting-State Instability and Rightward Parietal Dysfunction in ADHD. Annu. Int. Conf. IEEE Eng. Med. Biol. Soc..

[17] Parlatini V., Itahashi T., Lee Y., Liu S., Nguyen T.T., Aoki Y.Y., Forkel S.J., Catani M., Rubia K., Zhou J.H. (2023). White matter alterations in Attention-Deficit/Hyperactivity Disorder (ADHD): A systematic review of 129 diffusion imaging studies with meta-analysis. Mol. Psychiatry.

[18] Safren S.A., Otto M.W., Sprich S., Winett C.L., Wilens T.E., Biederman J. (2005). Cognitive-behavioral therapy for ADHD in medication-treated adults with continued symptoms. Behav. Res. Ther..

[19] Stevens J.R., Wilens T.E., Stern T.A. (2013). Using stimulants for attention-deficit/hyperactivity disorder: Clinical approaches and challenges. Prim. Care Companion CNS Disord..

[20] Adler L.A., Faraone S.V., Spencer T.J., Berglund P., Alperin S., Kessler R.C. (2017). The structure of adult ADHD. Int. J. Methods Psychiatr. Res..

[21] Ledbetter M. (2006). Atomoxetine: A novel treatment for child and adult ADHD. Neuropsychiatr. Dis. Treat..

[22] Evans S.W., Owens J.S., Bunford N. (2014). Evidence-based psychosocial treatments for children and adolescents with attention-deficit/hyperactivity disorder. J. Clin. Child Adolesc. Psychol..

[23] Ravishankar V., Chowdappa S.V., Benegal V., Muralidharan K. (2016). The efficacy of atomoxetine in treating adult attention deficit hyperactivity disorder (ADHD): A meta-analysis of controlled trials. Asian J. Psychiatry.

[24] Spencer T., Biederman J., Wilens T., Faraone S., Prince J., Gerard K., Doyle R., Parekh A., Kagan J., Bearman S.K. (2001). Efficacy of a mixed amphetamine salts compound in adults with attention-deficit/hyperactivity disorder. Arch. Gen. Psychiatry.

[25] Heal D.J., Smith S.L., Gosden J., Nutt D.J. (2013). Amphetamine, past and present—A pharmacological and clinical perspective. J. Psychopharmacol..

[26] Solanto M.V., Marks D.J., Mitchell K.J., Wasserstein J., Kofman M.D. (2008). Development of a new psychosocial treatment for adult ADHD. J. Atten. Disord..

[27] Nasri B., Castenfors M., Fredlund P., Ginsberg Y., Lindefors N., Kaldo V. (2020). Group Treatment for Adults With ADHD Based on a Novel Combination of Cognitive and Dialectical Behavior Interventions: A Feasibility Study. J. Atten. Disord..

[28] Nasri B., Cassel M., Enhärje J., Larsson M., Hirvikoski T., Ginsberg Y., Lindefors N., Kaldo V. (2023). Internet delivered cognitive behavioral therapy for adults with ADHD—A randomized controlled trial. Internet Interv..

[29] Enriquez-Geppert S., Smit D., Pimenta M.G., Arns M. (2019). Neurofeedback as a Treatment Intervention in ADHD: Current Evidence and Practice. Curr. Psychiatry Rep..

[30] Leffa D.T., Grevet E.H., Bau C.H.D., Schneider M., Ferrazza C.P., da Silva R.F., Miranda M.S., Picon F., Teche S.P., Sanches P. (2022). Transcranial direct current stimulation vs sham for the treatment of inattention in adults with attention-deficit/hyperactivity disorder: The TUNED randomized clinical trial. JAMA Psychiatry.

[31] Nasser A., Hull J.T., Chaturvedi S.A., Liranso T., Odebo O., Kosheleff A.R., Fry N., Cutler A.J., Rubin J., Schwabe S. (2022). A Phase III, Randomized, Double-Blind, Placebo-Controlled Trial Assessing the Efficacy and Safety of Viloxazine Extended-Release Capsules in Adults with Attention-Deficit/Hyperactivity Disorder. CNS Drugs.

[32] Alam N., Choudhary K. (2023). Neurochemical effects of methylphenidate and modafinil in ameliorating stress-induced cognitive deficits. ACS Pharmacol. Transl. Sci..

[33] Faraone S.V., Perlis R.H., Doyle A.E., Smoller J.W., Goralnick J.J., Holmgren M.A., Sklar P. (2005). Molecular genetics of attention-deficit/hyperactivity disorder. Biol. Psychiatry.

[34] Demontis D., Walters R.K., Martin J., Mattheisen M., Als T.D., Agerbo E., Baldursson G., Belliveau R., Bybjerg-Grauholm J., Bækvad-Hansen M. (2019). Discovery of the First Genome-Wide Significant Risk Loci for ADHD. Nat. Genet..

[35] Ronald A., de Bode N., Polderman T.J.C. (2021). Systematic Review: How the Attention-Deficit/Hyperactivity Disorder Polygenic Risk Score Adds to Our Understanding of ADHD and Associated Traits. J. Am. Acad. Child Adolesc. Psychiatry.

[36] Attention Deficit Hyperactivity Disorder: Diagnosis and Management; National Institute for Health and Care Excellence (NICE): London, UK, 2019; (NICE Guideline, No. 87). https://www.ncbi.nlm.nih.gov/books/NBK493361/.

[37] Antshel K.M., Hargrave T.M., Simonescu M., Kaul P., Hendricks K., Faraone S.V. (2011). Advances in understanding and treating ADHD. BMC Med..

[38] Sacco R., Camilleri N., Eberhardt J., Umla-Runge K., Newbury-Birch D. (2024). A systematic review and meta-analysis on the prevalence of mental disorders among children and adolescents in Europe. Eur. Child Adolesc. Psychiatry.

